# Design and Development of a Telerehabilitation Self-Management Program for Persons with Chronic Lower Limb Swelling and Mobility Limitations: Preliminary Evidence

**DOI:** 10.1155/2012/608059

**Published:** 2012-11-27

**Authors:** Becky L. Faett, Mary Jo Geyer, Leslie A. Hoffman, David M. Brienza

**Affiliations:** ^1^Department of Rehabilitation Science and Technology, School of Health and Rehabilitation Sciences, University of Pittsburgh, Suite 401, 6425 Penn Avenue, Pittsburgh, PA 15206, USA; ^2^Department of Acute and Tertiary Care, School of Nursing, University of Pittsburgh, 336 Victoria Building, 3500 Victoria Street, Pittsburgh, PA 15261, USA

## Abstract

This paper describes design and development of a self-management program, delivered by telerehabilitation (TR), to address the problem of chronic lower limb swelling in persons with limited mobility. The 18.6 million persons with limited mobility in the USA are at increased risk for chronic lower limb swelling and related secondary complications, including cellulitis and skin ulcers. Over time, chronic swelling often progresses to lymphedema, an incurable condition requiring lifelong care. Without successful self-management, lymphedema and its related complications inevitably worsen. Access and adherence to appropriate treatment are challenging for persons with limited mobility. Program development involved a structured process to establish content validity (videos and manuals), readability, suitability, and selection of a TR platform to deliver the educational program. Our goal was to develop a program that would engage patients in self-management skills. The TR software platform chosen, Versatile and Integrated System for Telerehabilitation (VISYTER) was designed to facilitate face-to-face delivery of an interactive home-based self-management program via the internet in real time. Results demonstrated validity of the educational program and ease of use with TR. Future plans are to evaluate ability of this approach to promote self-management skills, home monitoring, and improved management of persons with lymphedema and limited mobility.

## 1. Introduction

Lymphedema, an incurable health condition, occurs when an impairment of the lymphatic system results in a lymphatic load that is greater than lymphatic transport capacity. The consequence is accumulation of protein-rich fluid in the interstitial space [1, 2]. Over time, this high concentration of protein stimulates an inflammatory response leading to fibrotic changes in subcutaneous tissue and hypertrophy of adipose tissue [3]. Protein-rich lymph provides a fertile medium for bacterial growth [3]. If untreated, lymphedema can progress, causing continued proliferation of fibrotic tissue, an increase in size of the affected limb, an increased risk for wounds [4, 5], life-threatening infections, loss of functional ability [6], and decreased quality of life [7, 8]. Lymphedema is classified as primary or secondary. Primary lymphedema is congenital and rare [9], while secondary or “acquired” lymphedema is common. Causes of secondary lymphedema include surgery for carcinoma that involves damage or dissection of lymph nodes, radiation therapy, trauma to lymph nodes or vessels, chronic infection, chronic venous insufficiency, tumors that obstruct the lymphatic flow, and/or a combination of the these conditions [6, 10, 11]. 

People with mobility limitations are at high risk for chronic lower extremity swelling. The return of fluids via the venous and lymphatic system is facilitated by muscle contractions of the legs. This physiologic mechanism is hindered or absent in people who use wheelchairs for mobility. Sitting for long periods of time applies pressure to capillaries and lymphatic vessels that also impedes flow. Poor body trunk alignment negatively affects the normal respiratory pump for the lymphatic system and further reduces lymphatic flow [12].

Complete decongestive therapy (CDT) is the gold standard treatment for management of chronic lymphedema [13–16]. The goal of CDT is to reduce swelling and tissue fibrosis, improve functional ability, prevent infections [13, 16], and stop the progression of the disease. CDT is provided in two phases. Phase I is completed within the clinical setting by a certified lymphedema therapist. It consists of manual lymphatic drainage (specialized massage) to facilitate central lymph flow and promote movement of the lymph out of the effective limb, multilayer short stretch compression bandaging of the extremity, diaphragmatic breathing and exercise to further enhance lymph flow, a low salt diet, and meticulous skin care [2, 9, 10, 13, 16–18]. Phase II involves the continued maintenance of these labor-intensive activities for life by the patient. Nonadherence with home maintenance has been shown to minimize the benefits of CDT [19].

Access to therapists specializing in CDT is challenging. First, there are few certified lymphedema therapists and those available are primarily located in urban areas. Patients with limited mobility may have difficulty obtaining transportation that accommodates their needs. Public transportation is often difficult to access or unavailable, especially in rural areas [20]. Most wheelchair users (82%) report that public transportation is difficult to access [21]. Costs are another concern. Most third-party payers limit the number of paid visits per year and require copayments. Only 43% of people with disabilities earn wages for employment and 17% report they have no health care insurance [22]. In order to address barriers faced by people with limited mobility in achieving access to treatment and management of chronic swelling, alternative treatment strategies need to be identified. One such alternative is the use of telerehabilitation to provide a remote self-management program.

Telerehabilitation (TR) has been defined as “the application of telecommunications, remote sensing and operation, and computing technologies to the delivery of medical rehabilitation services at a distance” [23, p. 115]. Considered a subspecialty of telehealth, TR can be provided using a variety of modes. These include “face-to-face” videoconferencing, telehomecare to coordinate in-home therapy and patient support, in-home patient telemonitoring, and teletherapy for exercise supervision by a remote therapist [24]. Selection of the most appropriate mode requires consideration of the needs of the patient population, equipment capabilities, available bandwidth, and clinician skills. 

TR has the potential to improve access to specialty care and provide services within the home environment, thereby meeting needs expressed by those with mobility limitations. Limitation in mobility impacts an estimated 18.6 million people in the USA [20]. Studies have shown TR to be a valid and reliable modality for assessment of patients with mobility limitations. TR applications range from determining appropriate wheelchair seating to management of skin ulcers to providing complex treatment modalities [25–28]. TR has been shown to increase functional mobility in patients after stroke, [29, 30] after knee arthroplasty, [31] with cerebral palsy, [32] and with multiple sclerosis [33]. Telecommunication has also been shown to be effective in providing remote self-management programs for chronic conditions, resulting in improved health care outcomes [34–36]. Patients have reported high levels of satisfaction with health care delivered by TR [31, 37, 38]. 

Self-management is defined as “an individual's ability to manage the symptoms, treatment, physical and psychosocial consequences and lifestyle changes inherent in living with a chronic disease” [39, p.563]. Lorig and Holman [40] describe five core skills for self-management of chronic conditions. The first is problem solving, that is, the ability to identify both problems and possible solutions. Decision-making is the second skill, that is, having the knowledge to make appropriate decisions in response to one's current status. The third skill is developing the ability to find and utilize appropriate resources, that is, the Internet, library, community resources, and support groups. The fourth skill is the ability to develop a true partnership with health care professionals. Patients must work in conjunction with health care providers to appropriately evaluate and monitor their responses to therapy, to know when to ask for help, and determine when to modify their care in order to meet their needs. The fifth and final skill is the ability to implement and evaluate a plan of self-care. Patients need to learn how to develop their own short-term measurable goals, evaluate the level of their success in meeting those goals, and determine when they need to modify or set new goals to achieve optimum self-management of their chronic condition [40].

Self-management theory is grounded in the expectation that increasing patients' belief in their ability to manage their illness will result in positive change and better health care outcomes [41]. This belief, known as perceived self-efficacy, is defined as “the confidence a person feels about performing a particular activity, including confidence in overcoming the barriers to performing that behavior” [42, p. 173]. According to Albert Bandura's Social Cognitive Theory of Self-Regulation, a person's perception of their self-efficacy will impact their decisions in life, their goals, and how they respond to adversity. The more competent a person believes themselves to be, the higher the goals they will establish and the more determined they will be to overcome adversity in meeting those goals [43]. Research has shown a positive correlation between self-efficacy and health care outcomes [44, 45], and high self-efficacy is a predictor of the success of behavioral interventions [46, 47]. Self-management programs have been shown to increase self-efficacy, improve health behaviors [45], improve healthcare outcomes [45, 48, 49], and decrease health care utilization [50, 51]. Therefore, the intent of “Telerehabilitation: Empowering You to Manage and Prevent Swelling” (TR-PUMPS) was to deliver a standardized educational program for self-management of chronic lower limb swelling, monitor health status, and assess the ability to perform required skills via real-time teleconferencing in a population of persons with limited mobility. 

In order to address barriers faced by people with limited mobility, an alternative treatment strategy was proposed and designed for remote delivery via TR. The TR-PUMPS educational program was developed as a critical component of a clinical trial to evaluate the effects of a TR for immobile individuals with chronic edema/lymphedema of the lower limbs (NIDRR Grant no. H133E090002). In the clinical trial, TR was chosen as a novel delivery method for a modified lymphedema treatment protocol including advanced pneumatic compression. A review of the literature revealed no previous studies of self-management strategies for swelling in this high-risk population with limited mobility. 

## 2. Methods

### 2.1. Content Development

Based on current best practice for treatment of chronic edema/lymphedema, a draft version of the educational protocol was developed with input from three certified lymphedema therapists. Based on current best practice for treatment of chronic edema/lymphedema, the script identified 10 learning goals central to self-management for chronic edema of the lower extremities (see Table 1). The next challenge was to develop evidence-based content for each of these steps at a suitable reading level.

### 2.2. Evaluating Readability

Studies have shown that patient educational materials often have a higher readability level than recommended by the American Medical Association (5th to 6th grade) [52–56]. Patient comprehension had been shown to increase when presented at a 5th-grade level [57]. Thus, after developing draft content for the educational program, the next step involved testing readability level. Formulas frequently used to evaluate the grade level of a written text include the *Flesh Reading Ease Scale, Flesch-Kincaid, Gunning Fog Index, SMOG Formula,* and *Fry Formula *[58]. These formulas use the number of polysyllable words and sentence length to calculate readability [58]. Therefore, all have a common limitation—they assume that more syllables increase reading difficulty. However, in reality, multisyllable words may be easier to comprehend.

Electronic software packages offer another option. Mailloux, et al. [59] performed a study to compare results obtained when using readability formulas with those obtained when using four software packages: *Corporate Voice, Grammatix IV, Microsoft Word for Windows, and RightWriter*. Results showed significant differences between the several formulas, but no significant difference in the means of overall grade levels produced by the *Corporate Voice, Grammatrix IV, and RightWriter *software programs.

Because of the variability in readability level that could result from these various tools, we followed the recommendations to use several formulas and software programs [58, 59]. The computer software *RightWriter* (Elite Minds, Inc.) was used to evaluate the readability level of the educational scripts. *RightWriter *employs two readability formulas, the *Flesch-Kincaid* and the *Gunning Fog Index* and also evaluates for active versus passive voice and jargon. A second evaluation was completed using the *Microsoft Word for Windows *readability program.

### 2.3. Video Development

Next, the final revised scripts were used to develop videos for the TR protocol. A video was developed for each of the 10 steps with the length of each video ranging from 1.5 to 11 minutes. The videos illustrated specific skills such as decongestive exercises and donning and doffing of compression garments. Each video was subdivided into individual skills or tasks to permit ease of locating and viewing during TR sessions. To establish content validity, the videos were viewed by eight additional board-certified lymphedema therapists who anonymously ranked each video on accuracy and completeness of the information as well as the clarity of the presentation. A 5-point Likert scale (5 = strongly agree to 1 = strongly disagree) was used to determine the therapists' level of agreement with descriptive statements about the content of each video (See Table 2).

When the TR program is operational, the videos will be available electronically via a portal that participants can access from their home computer. The videos will also be used during teleconferencing to teach the 10-step program and to reinforce teaching during review sessions.

### 2.4. Educational Booklet

To supplement the videos, an educational booklet was developed using the same 10-step approach. Illustrations and still frames from the videos were used to augment the script. The booklet was evaluated for suitability utilizing the Suitability Assessment of Materials (SAM) [60, 61]. Suitability refers to the ability of the material to be understood and acceptable by the targeted patient population [60]. The SAM rates educational material using 6 criteria: (1) content, (2) literacy demand, (3) graphics, (4) layout and typography, (5) learning stimulation, and (6) motivation and cultural appropriateness. These criteria are comprised of 22 factors. Each factor is scored superior (2 points), adequate (1 point), or not suitable (0 point). Scores are totaled for an overall rating expressed as a percentage (actual score divided by total possible score). Educational material with a score of 0%–39% is given a rating of “inadequate suitability”, 40%–69% is given a rating of “adequate suitability”, and 70% and above is rated as “superior suitability” [60, 61].

### 2.5. Implementing Telerehabilitation Protocol

The* Versatile and Integrated System for Telerehabilitation* (VISYTER) software platform was chosen for use in this self-management program and tailored to specific educational needs. VISYTER was developed at the University of Pittsburgh, School of Health and Rehabilitation Sciences, Department of Rehabilitation Science and Technology with funding from the National Institute for Disability and Rehabilitation Research via the Rehabilitation Engineering and Research Center on Telerehabilitation. It was selected because of its demonstrated effectiveness and flexibility in conducting remote face-to-face evaluations [25]. VISYTER is a secure system that provides users the ability for real-time teleconferencing with multiple remote camera control, sharing of educational videos through Microsoft Windows Media Player, and the ability to archive teleconferencing sessions and still images [62]. Minimum computer requirements include “Pentium Dual Core processor 2 GHz with 2 GB of RAM and an NVIDIA GeForce 4 Series graphic card” [63]. VISYTER requires an internet connection with an upstream and downstream speed of approximately 1.5 Mbps for medium quality video.

**Figure 1:**
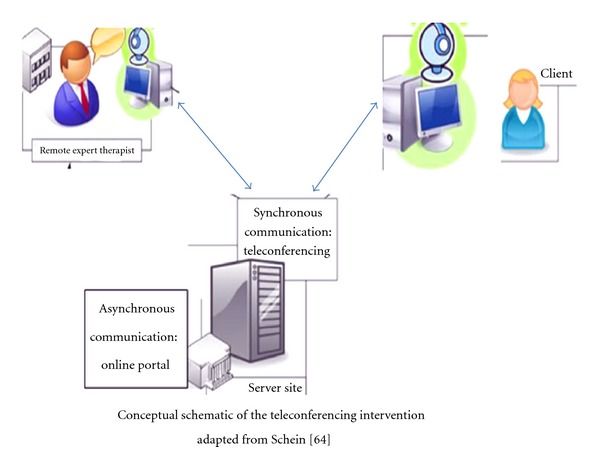
Conceptual schematic of teleconferencing intervention adapted with permission from Schein [64].

The use of the VISYTER system was tailored for use in the delivery of educational materials by loading the videos onto the system and performing laboratory testing of all capabilities prior to actual participant use. The first step when used in the home involves downloading the software onto a participant's home computer. Participants will be provided with a ClearOne CHAT 60 speakerphone. If no built-in web cam is available, a Logitech HD C910 web cam will be used for face-to-face videoconferencing. A second camera, the Logitech Orbit AF, can be connected to the participant's computer for use in skin and skill assessments. The Logitech Orbit AF will provide clinicians with the ability to remotely control the camera's movement and zoom capabilities. The clinician at the remote computer site will use a Logitech HD C910 web cam and a Logitech USB headset. A personal user ID and password will be assigned to each participant to enable them to log on to VISYTER. Each participant will also be assigned his or her own virtual clinic room. VISYTER's virtual clinic rooms are housed on the server and can only be accessed by authorized users. An assigned room administrator will determine the user's access and specific access capabilities which might vary depending upon their role [62]. Training will be provided to participants on how to use the VISYTER software to connect to the remote clinician (see Figure 1).

**Figure 2:**
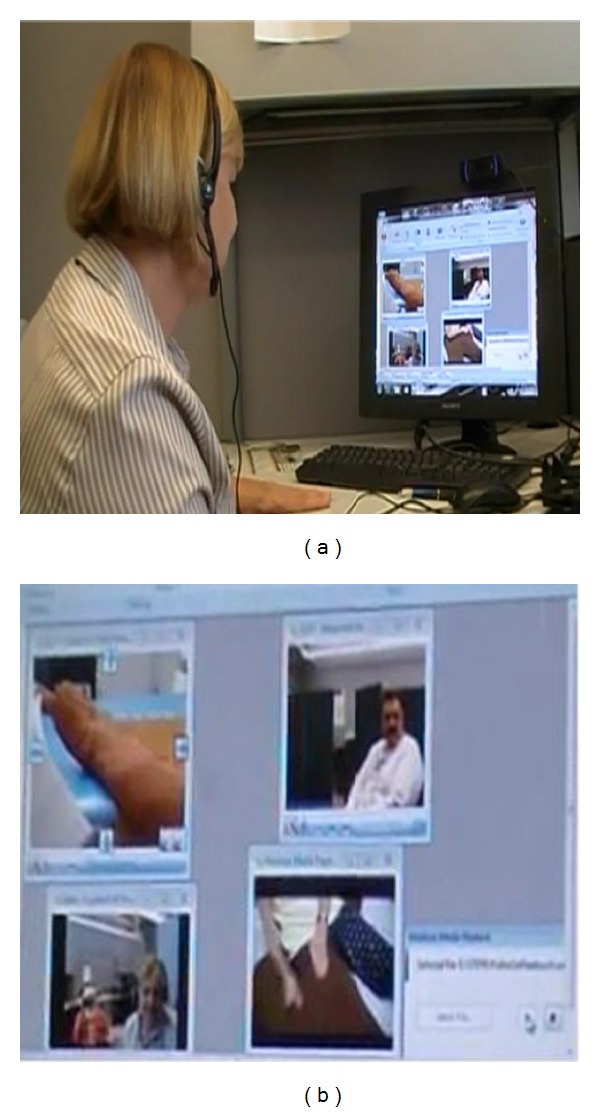
Remote clinician (a). Simulated telerehabilitation session (b). The right panel, top left, shows an image from video camera with remote control capabilities used for assessment; the right panel, top right, shows an image of a face-to-face intervention; the right panel, bottom left, shows an image of what the participant observes from their camera; the right panel, bottom right, shows educational video used for client education.

To implement the self-management program, the participant and clinician will connect to a VISYTER virtual clinic room via the portal in what is termed the “Lymphedema Venue” for each teleconferencing session. During the initial session, participants will be asked to identify five specific occupational performance goals. A plan of action will be developed in a collaborative effort between the participant and clinician. The participant's comprehension on lymphedema self-management will be evaluated, and any barriers that could negatively affect the participant's ability to perform the 10 steps of the self-management program will be identified (See Figure 2).

Real-time interactive education and evaluation sessions will occur a minimum of once per week for six weeks. The frequency of sessions will be determined by mutual agreement based on a joint review of the participant's current knowledge of their condition and self-management skills. Educational videos will be used to train the participants using the media sharing capabilities of the VISYTER software. Comprehension and retention of the educational material will be evaluated through participant verbal recall and performance of return demonstrations. All electronic communications will be encrypted to maintain privacy. TR interventions can be recorded and saved in an archived data base within the server.

The final step involved uploading the videos to the Lymphedema venue and the testing of the VISYTER software's performance in the delivery of the educational protocol within a laboratory setting. To conduct this evaluation, we transmitted from our virtual clinic workstation to distant rooms to test transmission in a simulated home setting. Bandwidth was tested to determine the speed necessary for providing the self-management program.

## 3. Results

### 3.1. Readability

When evaluated for readability utilizing the computer software* RightWriter* (Elite Minds, Inc.), *Flesh Kincaid* results indicated a readability level of a 4.01 grade level. The *Gunning Fog Index *resulted in readability level of a 5.61-grade level. The average of these two formulas resulted in an overall readability level below the 5th-grade level. A second evaluation using the *Microsoft Word for Windows *readability program resulted in a *Flesh Kincaid* readability level at a 3.4-grade level.

### 3.2. Content Validity

When content validity of the educational videos was determined by eight experienced, board-certified lymphedema therapists, the mean score was 4.5 ± 0.35 with a range of 4.1–4.9 (see Table 3).

### 3.3. Suitability

The supplemental educational booklet, developed using the same script as the videos, was evaluated for suitability utilizing the Suitability Assessment of Materials [61]. A score of 77% was achieved, resulting in a superior rating.

### 3.4. TR Program Function

The video file format, *QuickTime Format*, used for the videos was not compatible with the VISYTER software platform. The video file format was therefore changed to the *Windows Media Video Format *to allow sharing of the videos during video conferencing. The ability for participants to access and download videos from the lymphedema portal was verified.

Transmission speed was determined using two cameras plus audio upload on the participants' side and one camera and media sharing and audio download on the clinician's side. It was determined that a medium-quality video was sufficient for the face to-face interaction. However, high video quality was required for the remote camera for visualization during skin assessments. Results of the laboratory testing determined that speeds of 1220 kbps upload and download were the minimum requirements and the speed of 1620+ kbps upload and download provided optimal teleconferencing audio and visual quality.

## 4. Discussion

The study's researchers' collaborative efforts resulted in the development of a comprehensive self-management program for patients with chronic lymphedema with appropriate readability and suitability scores that was highly rated by external evaluators in regard to its content validity and selection of a software platform with the potential to provide in-home teleconferencing and assessment capabilities.

Healthy People 2010 defines health literacy as “the degree to which individuals have the capacity to obtain, process, and understand basic health information and services needed to make appropriate health decisions” [65]. The 2003 National Assessment of Adult Literacy (NAAL) survey results showed that only 12% of adults in the USA had proficient health literacy [66]. Inadequate health literacy has been associated with a decreased ability to communicate with health care providers [67], decreased knowledge and self-management skills of chronic conditions [68–70], and poorer health care outcomes [71, 72]. In order to address health literacy disparity, health care professionals need to provide education materials that are appropriate for all health literacy levels. Adult readers of all reading levels prefer and learn better with easy-to-read instructions [61]. Self-management programs that are tailored for patients with inadequate health literacy have been shown to overcome learning barriers, increase self-management skills, and reduce the rate of hospitalization and death [49, 73].

A variety of readability software and formulas are available to evaluate readability levels. A concern, as shown in our study, is that different readability formulas can produce discrepancies in grade-level results with scores deviating as high as 41% [59]. Consequently, we advise using several formulas and averaging the results [58, 59]. Our study produced results using several tools that ranged from 3.4 to 5.61, supporting findings of prior studies [58, 59]. To obtain additional information on readability formulas, the website for the* plain and simple *project from the Iowa Department of Public Health is recommended and may be accessed at http://www.idph.state.ia.us/PlainAndSimple/Readability.aspx. This website provides information on commonly used readability formulas and access to readability calculating software at no cost.

Readability level is not the only factor that determines the effectiveness of written material when attempting to address health literacy disparity [55, 61, 74]. Suitability of the material for the targeted population also needs to be evaluated. To perform this assessment, we chose the Suitability Assessment of Materials (SAM) developed by Cecila Doak and Jane Root. SAM was validated in a study enrolling 172 health care professionals from various countries [56] and has been widely used to evaluate educational material related to a variety of diseases [56, 75–78]. Additional information on *SAM* may be found in *Teaching Patients with Low Literacy Skills (2nd Ed)* by Doak et al. [61]. This text may be downloaded at no cost from http://www.hsph.harvard.edu/healthliteracy/resources/.

With advances in information technology, telecommunications offers health care providers the opportunity to utilize new approaches to health care delivery. The current health care delivery system is focused on acute care. With the aging population and the increase in chronic conditions, this health care delivery system is not meeting the public's needs. Chronic conditions result in seven out of ten deaths in the USA. As of 2005, 133 million people in the USA suffered from at least one chronic condition. This number is projected to increase to 157 million people by 2020 [79]. The increase in the prevalence of chronic conditions is contributed to the increasing age of the population and an increase in risk factors such as obesity which predispose chronic illness [79]. The occurrence of people having multiple chronic conditions increases with age, with 25% of Medicare recipients having at least four chronic conditions [41]. This rise in chronic conditions is creating an increased financial burden on the health care industry with costs associated with the management of these chronic diseases accounting for 75% of all health care costs [80]. A redesigning of the current health care system to provide a continuum of health care to people with chronic conditions is necessary [81]. Recommendations for redesign of health care delivery systems by the Institute of Medicine (IOM) include that patients be informed decision makers in their health care, health care should be customized according to patients' needs and values, and health care should be readily available and provided not just by face-to-face visits but also by internet or telephone [81]. In 2007, only 13.6% of people reported using the internet as a source of communication with their health care provider [82]. A goal of Healthy People 2020 is to increase this to 15.7% [82]. The utilization of TR to provide self-management programs for chronic conditions can be a potential resource in facilitating a continuum of care that is patient-centered and focused on providing patients with the knowledge and skills to enable them be actively involved in the management of their chronic conditions.

## 5. Conclusion

The evidence-based educational materials developed as part of the self-management program for lower limb chronic swelling/lymphedema in persons with limited mobility were found to be valid, accurate, and complete with high ratings of clarity. The readability and suitability ratings indicated that the materials are appropriate for various levels of health literacy in the study population. The self-management program described may be applicable to address other chronic conditions such as diabetes, heart failure, and chronic obstruction pulmonary disease. We believe TR is a viable method of providing a home-based self-management program on lower limb chronic swelling/lymphedema in people with mobility limitations and can decrease the burden associated with lifelong management of this debilitating condition.

## Figures and Tables

**Table 1:** 10 Steps to healthier feet and legs.

Step	Goal
1	Understand basic pathophysiology of the vascular and lymphatic system, including warning signs of vascular, lymphatic, and neurological damage.

2	Demonstrate proper limb hygiene, for example, washing and drying technique and nail care.

3	Describe proper use of moisturizers and appropriate application.

4	Relate steps during skin inspection for changes such as redness, wounds, skins cracks, blisters, and excessive dryness and increased hardness.

5	Describe proper care for minor skin wounds.

6	Describe how to select appropriate footwear and clothing.

7	Demonstrate strategies to prevent/minimize swelling, including leg elevation, avoidance of excessive heat, and proper diet.

8	Demonstrate appropriate deep breathing and decongestive exercises.

9	Demonstrate appropriate application and care of an advanced pneumatic compression device and compression garments.

10	State signs and symptoms and appropriate action when complications develop, for example, deep venous thrombosis, pulmonary emboli, pulmonary congestion, edema, and/or infection.

**Table 2:** Excerpts from the tool used to evaluate video content.

Please respond to the following questions by placing a number in the box that matches your level of agreement	Strongly agree	Agree	Neither agree nor disagree	Disagree	Strongly disagree
	5	4	3	2	1
Step 1: Know about your feet and legs					
(1) The basic anatomy and physiology are clearly explained					
(2) Anatomy and physiology provide rationale for interventions at basic level					
(3) The warning signs of poor circulation are accurate and complete					
(4) The warning signs for chronic swelling are accurate and complete					
(5) The warning signs for neuropathy are accurate and complete					
(6) Comorbidity is explained as well as the need for differential diagnosis					

Step 2: Wash and dry daily					
(1) Rationale for daily washing is clearly explained					
(2) Preparation for washing is accurate and complete					
(3) Washing technique is accurate and complete					
(4) Criteria for helper assistance and performance requirements are clearly explained					
(5) Preparation for drying is accurate and complete					
(6) Drying techniques are accurate and complete					
(7) Precautions are accurate and complete					
(8) Instructions to wash and dry both affected and unaffected foot and leg in the same manner are clear					

**Table 3:** Rating of lymphedema therapists for video content validity. Rating scale was 5: strongly agree to 1: strongly disagree.

Video	1	2	3	4	5	6	7	8	9	10
Mean	4	4.19	4.8	4.5	4.6	4.55	4.48	4.38	4.34	4.9
Mode	4	4	5	4	4	4	4	4	4	4
